# Prevalence and Assessment of Habits Related to Gastroesophageal Reflux Disease Among the Residents of Southwest Region in Saudi Arabia

**DOI:** 10.7759/cureus.63248

**Published:** 2024-06-26

**Authors:** Omar A Alshaikhi, Mohamed E Salih, Atyaf A Almarhabi, Hadeel A Alzubaidy, Amwaj A Alemshani, Shereen M Alamri, Nourah I Alzubaidi, Alaa F Samandar, Hassan A AlZubaidi, Mohammed J Himmat, Saleh A Alshaikhi

**Affiliations:** 1 Department of Pediatrics, Ministry of Health, Jeddah, SAU; 2 Department of Surgery, Al-Qunfudah Medical College, Umm Al-Qura University, Al-Qunfudah, SAU; 3 General and Colorectal Surgery, Wad-Medani Hospital, Medani, SDN; 4 Department of Medicine and Surgery, Al-Qunfudah Medical College, Umm Al-Qura University, Al-Qunfudah, SAU; 5 Department of Family and Community Medicine, King Abdulaziz Medical City, National Guard Health Affairs, Jeddah, SAU; 6 Department of Medicine and Surgery, Hayatt University College, Khartoum, SDN; 7 Department of Family Medicine, Ministry of Health, Jeddah, SAU

**Keywords:** awareness, alqunfudh, local habits, digestive disease, gastroesophageal reflex disease

## Abstract

Introduction

Digestive disorders, affecting 70 million people globally, have gained attention. Gastroesophageal reflux disease (GERD) causes heartburn and acid regurgitation. Factors like obesity, poor diet, and lifestyle influence its prevalence. GERD rates are higher in Saudi Arabia than in Western and East Asian countries, highlighting the need for local awareness, our study aims To assess the prevalence of local habits and risk factors of patients with GERD in the Southwest region of Saudi Arabia.

Method

This prospective descriptive cross-sectional online-based study included 372 individuals from the Southwest Region, Saudi Arabia. The study was conducted using a self-administrated online questionnaire to collect the data for the targeted participants. After obtaining consent to carry out the study, the data were collected and computerized using Microsoft Excel, to form a database using all the items from the data collecting sheets. Data was then encoded and analyzed using Statistical Package for Social Sciences (SPSS) software, version 27 (IBM Corp., Armonk, NY). Data was displayed and presented in the form of tables and charts

Results

This study included 372 individuals, predominantly Saudi 98.7%, n=367. The most common age group was 21-25 years (32.5%, n=121), followed by those over 40 (18.5%, n=69). Participants were mainly from Al-Qunfudhah and Haly 27.2%, n=101. Most were single (49.5%, n=184) or married (48.4%, n=180), with a bachelor's degree 66.7%, n=248. Employees comprised (36.3%, n=135), and students 30.9%, n=115. Only 2.7%, n=10 were pregnant. Nearly half (48.4%, n=180) had a monthly income below 5,000 SAR. Most participants weighed 40-60 kg (41.4%, n=154) and were 150-160 cm tall (47%, n=175). The prevalence of GERD was 16.1%, n=60. Significant associations were found between GERD diagnosis and meal type, smoking, family history, and high pickle and salt intake (P-values: 0.002073, 0.000607, <0.00001, and 0.008557, respectively).

Conclusion

This study can conclude that the prevalence of GERD is not high in the Southwest region of Saudi Arabia. Significant risk factors regarding patients’ habits should be taken into consideration and diminishing them in order to decrease the incidence of the disease and improve the quality of life of already diagnosed patients.

## Introduction

In the past few years, digestive disorders have become an important subject and a great matter of interest worldwide, where an estimated 70 million people suffer from them [1]. Although Gastroesophageal reflux disease (GERD) is considered one of the most common gastrointestinal diseases in adults [2], it is described as a physiological backflow of the gastric contents into the esophagus. It results in uncomfortable symptoms such as heartburn and/or acid regurgitation and injury of the esophageal mucosa [3].

GERD can be divided into three sub-types based on the symptomatic manifestations of the disease [4]. It is characterized by diverse symptoms and signs, including heartburn, difficulty & painful swallowing, abdominal and epigastric pain, nausea, vomiting of blood, and weight loss [5], which can have a negative impact on the quality of life of the patients [6]. GERD risk factors include obesity, junk food, lack of physical activity, smoking, alcohol, and high sugar intake [7,8].

A Polish study identified a correlation between the severity of typical GERD symptoms and specific dietary habits. Symptoms were more prevalent after consuming fatty, fried, sour, or spicy foods, as well as sweets including peppermint tea [9]. A Saudi study has revealed a higher prevalence of GERD in Saudi Arabia compared to Western countries and much higher than in countries of East Asia [10]. Another Saudi study has shown a lot of variables associated with symptomatic GERD such as smoking, gender, family history, obesity, and sleeping immediately after dinner or within 1 hour. Therefore, it is important to address the local public awareness of the risk factors [11]. A different Saudi study among the adults attending Primary health care in Abha City, Saudi Arabia, showed the prevalence of GERD to be 67.8%, which directly impacts the lives of those affected [12].

The COVID-19 pandemic played a great role in understanding GERD and its risk factors where the quarantine had affected people's habits regarding food and sports as a study showed a higher prevalence compared to the pre-pandemic period. The majority of individuals exposed to these risk factors reported experiencing GERD symptoms [13,14]. GERD can also affect different ages and related to certain dietary habits as revealed in Jazan University study [15].

While different studies were conducted to study the risk factors of GERD, the aim of this study was to further our understanding of the prevalence of GERD and investigate the relationship between local habits and GRED. GERD is a prevalent upper gastrointestinal disorder characterized by heartburn and acid regurgitation. A greater incidence is found in Arab countries. Untreated GERD can have a negative effect on patients, restrict daily activities, and impair their quality of life. This study is meant to fill the gap in research on the prevalence, local habits, and risk factors of GERD in the Southwest region of Saudi Arabia. To the best of our knowledge, no studies were conducted in this region to assess this matter.

## Materials and methods

Objectives

The objective of this study was to assess the prevalence, local habits, and risk factors of patients with GERD in the Southwest region of Saudi Arabia.

Study design

The study was conducted as a prospective descriptive cross-sectional online-based study in the Southwest Region of Saudi Arabia. It targeted the general population by utilizing convenient random sampling and an estimated sample size of 372. Male and female adults aged 15 years and more living in the Southwest Region of Saudi Arabia who accepted to participate in this study were included in this study; otherwise, they were excluded.

Data collection tools

The data was collected through an online questionnaire using e-mails and other social media platforms, including WhatsApp, X, and Telegram. A cover page that illustrated both the purpose of the study and the participant's consent was used. The questionnaire included four sections, the first one contained the socio-demographic characteristics of the participants such as gender, age, nationality, residence, marital status, educational level, monthly income, occupation, body weight, height, etc. While the second section had two questions to assess the general knowledge of participants, and three questions to assess the prevalence and the confirmation of the disease. The third section contained 15 questions to assess the local habits, risk factors and complains of participants.

Plan for data analysis

After obtaining consent to carry out the study, the data were collected and computerized using Microsoft Excel, to form a database using all the items from the data collecting sheets. Data was then encoded and analyzed using Statistical Package for Social Sciences (SPSS) software, version 27 (IBM Corp., Armonk, NY). Data was displayed and presented in the form of tables and charts.

Ethical consideration

Ethical approval was sought from Umm Al-Qura University with approval number HAPO-02-K-012-2024-02-2018 before starting the study. The objectives and benefits of the study were explained to the participants. Confidentiality and privacy of participants were maintained. The participants had the right to withdraw consent at any time without any consequences.

## Results

Three hundred and seventy-two individuals were included in this study. The most common age was found to be 21-25 years (32.5%, n=121), followed by more than 40 years (18.5%, n=69), and the majority (98.7%, n=367) were Saudi. The regions with the most participants were both Al-Qunfudhah and Haly (27.2%, n=101). Of the total participants, 49.5%, n=184, were single, while 48.4%, n=180 were married. The majority (66.7%, n=248) had a bachelor’s degree, followed by a secondary school degree (23.4%, n=87). 36.3%, n=135 were employees, followed by students (30.9%, n=115). Only 2.7%, n=10 were pregnant. Almost half of the respondents (48.4%, n=180) had a monthly income of less than 5.000, followed by 5.000-10.000 (15.9%, n=59) (Table 1).

**Table 1:** Sociodemographic characteristics of respondents All values are presented in numbers and percentages.

Variables	Category	Count	Percentage
Age	15-20 years	48	12.9%
	21-25 years	121	32.5%
	26-30 years	46	12.4%
	31-35 years	41	11%
	36-40 years	47	12.6%
	More than 40 years	69	18.5%
Nationality	Saudi	367	98.7%
	Non-Saudi	5	1.3%
Region	Al Qunfudhah	101	27.2%
	AlQouz	76	20.4%
	Almuzaylif	39	10.5%
	Jeddah	10	2.7%
	Hafar Al Batin	5	1.3%
	Haly	101	27.2%
	Enaker	6	1.6%
	Other	34	9.1%
Marital status	Single	184	49.5%
	Married	180	48.4%
	Divorced	4	1.1%
	Widow	4	1.1%
Educational level	None	10	2.7%
	Primary school	1	2.3%
	Secondary school	87	23.4%
	High school	8	2.2%
	Bachelor’s degree	248	66.7%
	Master degree	13	3.5%
	P.H degree	5	1.3%
Occupation	Unemployed	67	18%
	Student	115	30.9%
	Housewife	41	11%
	Employee	135	36.3%
	Healthcare employer	5	1.3%
	Retired	9	2.4%
Are you pregnant?	Yes	10	2.7%
	No	362	97.3%
Monthly income	Less than 5.000	180	48.4%
	5.000-10.000	59	15.9%
	10.000-15.000	50	13.4%
	15.000-20.000	47	12.6%
	More than 20.000	36	9.7%
Total		372	100%

Of the total participants, 41.4%, n=154 had a weight of 40-60 kg, followed by 60-80 kg (32.3%, n=120), while 47%, n=175 had a height of 150-160 cm, followed by 160-170 cm (23.7%, n=88) (Table 2).

**Table 2:** Weight and height of respondents All values are presented in numbers and percentages.

Variables	Category	Count	Percentage
Weight	Less than 40 kg	47	12.6%
	40-60 kg	154	41.4%
	60-80 kg	120	32.3%
	80-100 kg	44	11.8%
	100-120 kg	5	1.3%
	More than 120 kg	2	0.5%
Height	Less than 150 cm	60	16.1%
	150-160 cm	175	47%
	160-170 cm	88	23.7%
	170-180 cm	45	12.1%
	180-190 cm	3	0.8%
	More than 190 cm	1	0.3%
Total		372	100%

The majority (87.1%, n=324) have heard of GERD, while 12.9%, n=48 have not. Social media (37.1%, n=138) was the most common source of information, followed by physicians and doctors (19.1%, n=71). The prevalence of GERD was found to be 16.1%, n=60. Of them, 40%, n=24, did an endoscopy at the time of diagnosis, while 60%, n=36, did not, and 81.7%, n=49, were prescribed medicines for GERD, while 18.3%, n=11, were not. 38.3%, n=23 were prescribed Ibuprofen, followed by Panadol (26.7%, n=16) (Table 3).

**Table 3:** The general knowledge and prevalence of GERD among participants All values are presented in numbers and percentages. GERD: Gastroesophageal reflux disease.

Variables	Category	Count	Percentage
Ever heard of GERD?	Yes	324	87.1%
	No	48	12.9%
How did You know or hear about GERD?	Social Media	138	37.1%
	Tv	8	2.2%
	Family	53	14.2%
	Friends	35	9.4%
	Internet	67	18%
	Physicians & Doctors	71	19.1%
Ever diagnosed with GERD?	Yes	60	16.1%
	No	312	83.9%
If yes, did you do an endoscopy at the time of diagnosis? (n=60)	Yes	24	40%
	No	36	60%
If yes, were you prescribed any medicines for GERD? (n=60)	Yes	49	81.7%
	No	11	18.3%
Total		372	100%

About habits regarding GERD, 31.7%, n=19, did physical activity for more than 30 minutes two to three times per week. The majority (60%, n=36) eat less than three meals, and regarding type of meals, 40%, n=24 are fatty meals, followed by spicy meals (33.3%, n=20). 45%, n=27 drink soft drinks followed by drinks with caffeine (31.7%, n=19). 15%, n=9 were smokers, while 85%, n=51 were not. The majority (56.7%, n=34) have a family history of GERD, while 43.3%, n=26 do not. The majority (55%, n=33) eat a lot of pickles and salt, and 76.7%, n=46 eat fast food. 38.3%, n=23 have heartburn for two to three days per week, followed by one day (31.7%, n=19). When asked about the number of days in which the stomach contents, whether food or other things, move up into the throat or mouth, the most common answers were both one day and two to three days (31.7%, n=19) each. Of the total, 31.7%, n=19, said they feel pain in the middle of the upper part of the stomach one day per week, followed by 2-3 days (30%, n=18). 36.7%, n=22 stated that they get nausea one day per week, followed by two to three days per week (30%, n=18). Regarding difficulty getting a good night's sleep due to heartburn and/or reflux, the most common answer was two to three days (40%, n=24), followed by one day (28.3%, n=17). 33.3%, n=20, said they do not take additional medications for heartburn and/or reflux other than what their doctor told them to take, while 28.3%, n=17 do two to three days per week (Table 4).

**Table 4:** Participant responses concerning habits regarding GERD All values are in numbers and percentages. GERD: Gastroesophageal reflux disease.

Variables	Category	Count	Percentage
Physical activity for more than 30 minutes per week.	Never	11	18.3%
	Once	16	26.7%
	2-3 times	19	31.7%
	More than 3 times	14	23.3%
Have you been prescribed with any medications?	Ibuprofen	23	38.3%
	Panadol	16	26.7%
	Other	1	1.7%
	No	20	33.3%
Number of meals per day.	Less than 3 meals	36	60%
	3 meals	21	35%
	More than 3 meals	3	5%
Type of meals.	Spicy	20	33.3%
	Fatty	24	40%
	Sugars	6	10%
	Healthy	10	16.7%
Drinks.	Drinks with caffeine	19	31.7%
	Sour drinks	8	13.3%
	Soft drinks	27	45%
	Other	6	10%
Smoking.	Yes	9	15%
	No	51	85%
Family history of GERD.	Yes	34	56.7%
	No	26	43.3%
Do you eat a lot of pickles and salt?	Yes	33	55%
	No	27	45%
Do you eat fast food?	Yes	46	76.7%
	No	14	23.3%
How many days do you have heartburn?	None	4	6.7%
	One day	19	31.7%
	2-3 days	23	38.3%
	4-7 days	14	23.3%
How many days did the stomach contents (whether food or other things) move up into the throat or mouth?	None	9	15%
	One day	19	31.7%
	2-3 days	19	31.7%
	4-7 days	13	21.7%
How many days have you felt pain in the middle of the upper part of the stomach?	None	9	15%
	One day	19	31.7%
	2-3 days	18	30%
	4-7 days	14	23.3%
How many days did you get nausea?	None	8	13.3%
	One day	22	36.7%
	2-3 days	18	30%
	4-7 days	12	20%
How many days have you had difficulty getting a good night's sleep due to heartburn and/or reflux?	None	6	10%
	One day	17	28.3%
	2-3 days	24	40%
	4-7 days	13	21.7%
How many days have you taken additional medications for heartburn and/or reflux, other than what your doctor told you to take?	None	20	33.3%
	One day	16	26.7%
	2-3 days	17	28.3%
	4-7 days	7	11.7%
Total		60	100%

According to Table 5, Significant associations were found between diagnosis of GERD and type of meals, smoking, family history of GERD, and eating a lot of pickles and salt (P-value = 0.002073, 0.000607, < 0.00001, and 0.008557, respectively).

**Table 5:** The correlation between diagnosis of GERD and risk factors All values are presented In numbers; P-values less than 0.05 were considered statistically significant. *: Significant P-value; GERD: Gastroesophageal reflux disease.

Variables	Category	Diagnosis of GERD		P-value
		Yes	No	
Physical activity for more than 30 minutes per week.	Never	11	87	0.269903
	Once	16	94	
	2-3 times	19	73	
	More than 3 times	14	58	
Number of meals per day.	Less than 3 meals	36	160	0.464336
	3 meals	21	133	
	More than 3 meals	3	19	
Type of meals.	Spicy	20	50	0.002073*
	Fatty	24	127	
	Sugars	6	21	
	Healthy	10	114	
Drinks.	Drinks with caffeine	19	149	0.118119
	Sour drinks	8	26	
	Soft drinks	27	107	
	Other	6	30	
Smoking.	Yes	9	12	0.000607*
	No	51	300	
Family history of GERD.	Yes	34	62	< 0.00001*
	No	26	243	
Do you eat a lot of pickles and salt?	Yes	33	115	0.008557*
	No	27	197	
Do you eat fast food?	Yes	46	246	0.706689
	No	14	66	

## Discussion

In a study carried out in Saudi Arabia by Al Ghadeer et al. in the year 2021, a total of 1517 participants were included, which is a bigger sample size than the one found in the current study (372 individuals) [16]. In this study, 58.8% were males, 41.2% were females, 9% were pregnant, and only (2.7%) were pregnant in the current study. The age of participants ranged from 18 to 58 years with a mean age of 27.5 ± 11.4 years, which is almost similar to the findings of the current study. The prevalence of GERD was 20.6% among the total participants, which is slightly higher than the one found in the current study (18.1%). The higher risk groups of having GERD were pregnant women, smokers, males, regular usage of analgesia, and soft drinks, and having a family history of GERD, while in the current study, the significant risk factors were the type of meals, smoking, family history of GERD, and eating a lot of pickles and salt [16]. 

When comparing the findings of the study conducted in Saudi Arabia by Alswat et al. in the year 2018 on the same topic. The sample was comprised of 2,043 participants, which is again, higher than the one found in the current study (372 individuals). Females and males were 51.8% and 48.2%, respectively. The mean age was 29.6 years with the standard deviation of 10.5 years. The GERD prevalence was 28.7%, which is also slightly higher than the one found in the current study (18.1%). It was found statistically significant among divorced/widows (34.9%, P = 0.003). However, in the current study, significant associations were found between diagnosis of GERD and type of meals, smoking, family history of GERD, and eating a lot of pickles and salt (P-value = 0.002073, 0.000607, < 0.00001, and 0.008557, respectively) [10].

In a study that was done in Syria by Shammout et al. in the year 2024 to investigate the prevalence of GERD symptoms, medication use, and impact on quality of life among students at a Syrian private university, 37.4% of the students reported not experiencing GERD symptoms, while the remaining did, with bloating (27.8%) being most prevalent, followed by sleep disturbances (22.2%), heartburn (21.5%), and regurgitation (18.3%) [17]. Only 16.8% used GERD medications such as proton pump inhibitors or antacids. Most students (68.5%) scored in the 0-15 range on the GERD-Health-Related Quality of Life (HRQL), indicating a minimal impact on quality of life. In this study, the prevalence of GERD was positively associated with age (P = 0.001), female sex (P = 0.001), and medical specialty (P = 0.0025). Whereas in the current study, the type of meals, smoking, family history of GERD, and eating a lot of pickles and salt were the significant risk factors for GERD [17].

In 2020, a study was carried out in Iran by Karimian et al. to investigate the epidemiology of GERD in Iran. The daily, weekly, monthly, and overall prevalence of GERD symptoms in the Iranian population was 5.64%, 12.50%, 18.62%, and 43.07%, respectively. The daily, weekly, monthly, and overall prevalence of heartburn in the Iranian population was 2.46%, 9.52%, 8.19%, and 23.20%, respectively. It can be noticed that the overall prevalence of GERD in the previously done study is slightly higher than the one found in the current study (18.1%). The daily, weekly, monthly, and overall prevalence of regurgitation in the Iranian population was 4.00%, 9.79%, 13.76%, and 36.53%, respectively. Whereas, the overall prevalence of regurgitation in the current study was found to be 85%, which is higher than the one found in the previous study [18].

Lastly, the study that was conducted in Saudi Arabia by Algethami et al. in the year 2018 to determine the prevalence and impact of GERD on the pilgrims in Mecca region during the Hajj period in the year 1438 Hegira showed a prevalence of GERD of 29.0% (again, is higher than the one found in the current study which is 18.1%), with a statistically significant association with age and nationality. Neither smoking nor the presence of other diseases showed a statistically significant relationship with the presence of GERD (P > 0.05). However, the current study showed a significant association with smoking and other risk factors such as type of meals, family history of GERD, and eating a lot of pickles and salt [19].

In summary, it was noticed that the results of the majority of the previously done studies align with the ones found in the current study regarding the prevalence of GERD, supporting the evidence and diminishing the gap found in the literature review. However, differences were found in other studies regarding the risk factors, which could be due to the targeted population (children/adults), or the differences in cultures and societies of different regions.

Recommendations

The findings of our study recommend providing effective measures on GERD-related factors such as lifestyle should be among the health policies and also advise that Healthcare providers should work on increasing the awareness of the public about the risk factors in order to avoid them, As well as proper counseling should be provided to already diagnosed patients, explaining the risk factors to them and stating the healthy habits for them to follow. More studies regarding this topic should be conducted in order to support the evidence and improve the quality of life.

Limitations

The study was conducted blindly on the general population without focusing only on diseased individuals. We had no access to gastroenterology clinics to interview the disease participants directly, so we depended on online questionnaires.

## Conclusions

This study can conclude that the prevalence of GERD is not high at the Southwest region of Saudi Arabia. Significant risk factors regarding patients’ habits should be taken into consideration and diminishing them in order to decrease the incidence of the disease and improve the quality of life of already diagnosed patients.
